# Single Inhalation of Peppermint Essential Oil Alleviates Acute Restraint Stress‐Exacerbated Itch in Oxazolone‐Induced Mild Dermatitis: Correlations With Brain Neuronal Activity in Female BALB/c Mouse

**DOI:** 10.1002/brb3.71072

**Published:** 2025-11-17

**Authors:** Shotaro Shoji, Yusuke Murata, Hikari Ikeda, Reika Sakai, Yuka Chijiiwa, Masayoshi Mori, Munechika Enjoji

**Affiliations:** ^1^ Department of Pharmacotherapeutics, Faculty of Pharmaceutical Sciences Fukuoka University Jonan‐ku Fukuoka Japan

**Keywords:** acute stress, itch‐related brain region, oxazolone‐induced dermatitis, peppermint essential oil

## Abstract

**Introduction**: Peppermint essential oil (PEO) and its main component, menthol, are used in Western and Eastern traditional medicine for their anti‐spasmodic, anti‐septic, or anti‐pruritic properties. Although topical application of PEO exhibits anti‐pruritic efficacy, the effects of PEO inhalation on itch sensation and pruritic behavior remain unclear. We aimed to determine whether PEO inhalation alleviates pruritus and itch‐responsive neural activity in the brains of mice with hapten‐induced dermatitis under acute stress conditions.

**Methods**: Forty‐one female BALB/c mice were randomly assigned to six experimental groups. Twenty‐nine mice were subjected to oxazolone (OXA)‐induced dermatitis through an initial sensitization followed by three rounds of topical application of OXA every other day. During the final OXA application, twenty‐two mice were exposed to restraint stress for 2 h. Sixteen mice were subjected to the inhalation of 2.5% PEO. The total duration of scratching bouts and the number of c‐Fos‐positive cells in the parabrachial nucleus, central amygdala, periaqueductal gray, and ventral tegmental area were quantified.

**Results**: PEO inhalation reduced the duration of scratching behavior induced by the combination of repeated OXA application and acute restraint stress. The c‐Fos‐positive cell number in the tested brain regions, except the ventral tegmental area, was positively correlated with the pruritic response. PEO inhalation alleviates OXA‐ or stress‐induced pruritus by modulating neuronal activity in itch‐related brain regions.

**Conclusion**: Acute restraint stress exacerbates itch, and PEO inhalation alleviates the stress‐associated aggravation of pruritus caused by OXA‐induced dermatitis, which is associated with the modulation of neuronal activity in itch‐related brain regions.

## Graphical abstract

Acute restraint stress increased the total duration of scratching behavior in mice regardless of whether dermatitis is present. The number of c‐Fos‐positive cells correlated with pruritic responses. Inhalation of peppermint essential oil alleviated OXA‐induced pruritus under stress conditions and increased the number of c‐Fos‐positive cells in the itch‐responsive brain region.

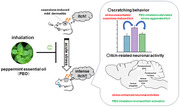

## 1 Introduction

Itch, or pruritus, is an unpleasant sensation that causes a desire to scratch (Ikoma et al. 2006). Exogenous pruritic stimuli, both mechanical and chemical, result in an itching sensation via the excitation of peripheral sensory nerve fibers distributed in the skin (Acton et al. 2019). The itch signals originating from peripheral itch stimuli or pathological skin conditions are relayed by peripheral sensory fibers to the spinal cord and then to the brain, inducing scratching. The itch‐scratch cycle is a vicious cycle in which chronic pruritus induces scratching, compromising the skin barrier and leading to inflammation and further itching (Tominaga and Takamori 2022). Hence, anti‐pruritic interventions are crucial for managing itch‐related diseases.

Itch signal processing is modulated by several physical and psychological factors (Mochizuki and Kakigi 2015). For example, chronic stress triggers or enhances itching, especially in pruritic dermatitis (Kim and Yosipovitch 2013), because psychological stress affects the neuroendocrine and immune systems and skin barrier functions (Arndt, Smith, and Tausk 2008; Urakami et al. 2024). Chronic itch, in turn, is a strong stressor (Passeron et al. 2021) that impairs the quality of life of individuals suffering from pruritus, leading to the increased risk of anxiety and depression (Lee et al. 2018; Marron et al. 2016; Matterne et al. 2013). Conventional dermatological care using pharmacological agents (e.g., oral anti‐histaminergic agents, topical anti‐inflammatory drugs, and systemic immunomodulators) has focused on local pathological symptoms, such as the dysfunctional epidermal barrier, skin inflammation, or pruritic behaviors (Nakagawa and Yamada 2023). Recent research on mice with hapten‐induced dermatitis has focused on developing novel therapeutics for pruritus, with an emphasis on approaches such as drug repositioning or utilization of plant‐derived materials (Habbas et al. 2025; Hussein et al. 2025). However, novel treatment strategies targeting the bidirectional and vicious relationship between itching and stress should be developed, because of that pruritus is associated with mental health problems.

Topical application of peppermint essential oil (PEO) and its primary constituent, menthol, is used in anti‐pruritic treatment (Gonçalves et al. 2024; Parvizi et al. 2022). However, it remains unclear whether the PEO inhalation affects itching sensation and pruritic behavior. The inhalation of aromatic essential oils, which is a frequently used application in aromatherapy, triggers a response in the brain via the olfactory system and modulates emotional responses (Lv et al. 2013). Hence, we hypothesized that the inhalation of PEO exerts a potential anti‐pruritic effect under itching or stressful conditions. Therefore, the primary aim of this study was to elucidate whether PEO inhalation reduces scratching behavior in mice with dermatitis under non‐stress or stress conditions.

Although the central mechanism governing itching responses in the brain remains unexplored, neural circuits underlying sensory, emotional, and motivational components of itch have been reviewed (Mu and Sun 2022). Itching is associated with negative emotion, and scratching induces pleasure and itch relief. Studies in rodents have shown that the parabrachial nucleus (PBN) is involved in pain and itch processing (Akiyama et al. 2016; Jansen and Giesler 2015; Li et al. 2021). Human brain imaging studies show the amygdala is activated by itching stimuli and deactivated by scratching (Papoiu et al. 2013; Vierow et al. 2015). Activating itch‐responsive central amygdala (CeA) neurons enhances scratching and anxiety‐like behaviors (Samineni et al. 2021; Sanders, Sakai, Henry, Hashimoto, and Akiyama 2019). Thus, itch‐induced discomfort may be processed via the CeA. Rodent studies showed pruritic stimuli activate the lateral external PBN subdivision projecting to the CeA (Sanders et al. 2019). The periaqueductal gray (PAG) regulates sensory and affective aspects of acute and chronic itching and modulates itch's affective components (Samineni et al. 2019). Dopaminergic innervations from the ventral tegmental area (VTA) to the nucleus accumbens influence motivation, and dopamine receptors affect itch‐evoked scratching behavior (Akimoto and Furuse 2011; Bromberg‐Martin, Matsumoto, and Hikosaka 2010). Although itch‐responsive brain regions regulate pruritic behavior, neuronal activity modulation in response to PEO inhalation remains unexplored. Therefore, the second objective was to elucidate whether PEO inhalation attenuates pruritus‐induced activation of itch‐responsive neurons under non‐stress or stress conditions. To this end, we used a mouse model exhibiting mild dermatitis and associated itching induced by short‐term application of the hapten oxazolone (OXA). We aimed to clarify whether a single PEO inhalation could suppress itching associated with OXA‐induced mild dermatitis and whether it could mitigate the acute stress‐induced exacerbation of itching.

## 2 Materials and Methods

### 2.1 Animals and Housing Conditions

Forty‐one specific‐pathogen‐free female BALB/c mice (7‐week‐old, weighing 19.3–21.5 g; Jackson Laboratory Japan Inc., Tokyo, Japan) were housed individually under these conditions: 12:12 h light‐dark cycle with lights on at 7:00 a.m., temperature of 23°C ± 2°C, and humidity of 60% ± 2%. Food and water were provided ad libitum. All animal experiments followed regulations established by the Experimental Animal Care and Use Committee of Fukuoka University and universal principles of laboratory animal care (approval numbers: 2204003 and 2304004; dates of approval: 4/19/2022 and 4/26/2023, respectively).

### 2.2 OXA‐Induced Mild Dermatitis Model

4‐Ethoxymethylene‐2‐phenyl‐2‐oxazolin‐5‐one (oxazolone [OXA], #862207) was used as a sensitizer to induce mild dermatitis in BALB/c mice according to a previously described method with minor modifications (Donglang et al. 2021). Briefly, OXA was dissolved in a mixture of acetone and olive oil at a ratio of 4:1 (vehicle). One day before sensitization, the abdomen and nape of the neck of the mice were shaved. The abdomen of mice was then sensitized by applying a 1.5% OXA solution (50 µL)‐impregnated patch for 1 h. After five days of sensitization, 20 µL of 0.5% OXA solution was applied three times to the nape of the neck every other day to induce dermatitis. Dermatitis induced by this protocol was mild and did not present characteristic symptoms such as erythema or edema; consequently, the severity score of dermatitis was not determined. Sham‐treated animals received the vehicle only.

### 2.3 Acute Restraint Stress

Restraint stress was induced by placing each mouse in an adequately ventilated 50 mL conical plastic tube for 2 h. The mice were not physically squeezed and experienced no pain. Control animals were moved to separate rooms and not subjected to stress.

### 2.4 Inhalation of PEO

**FIGURE 1:**
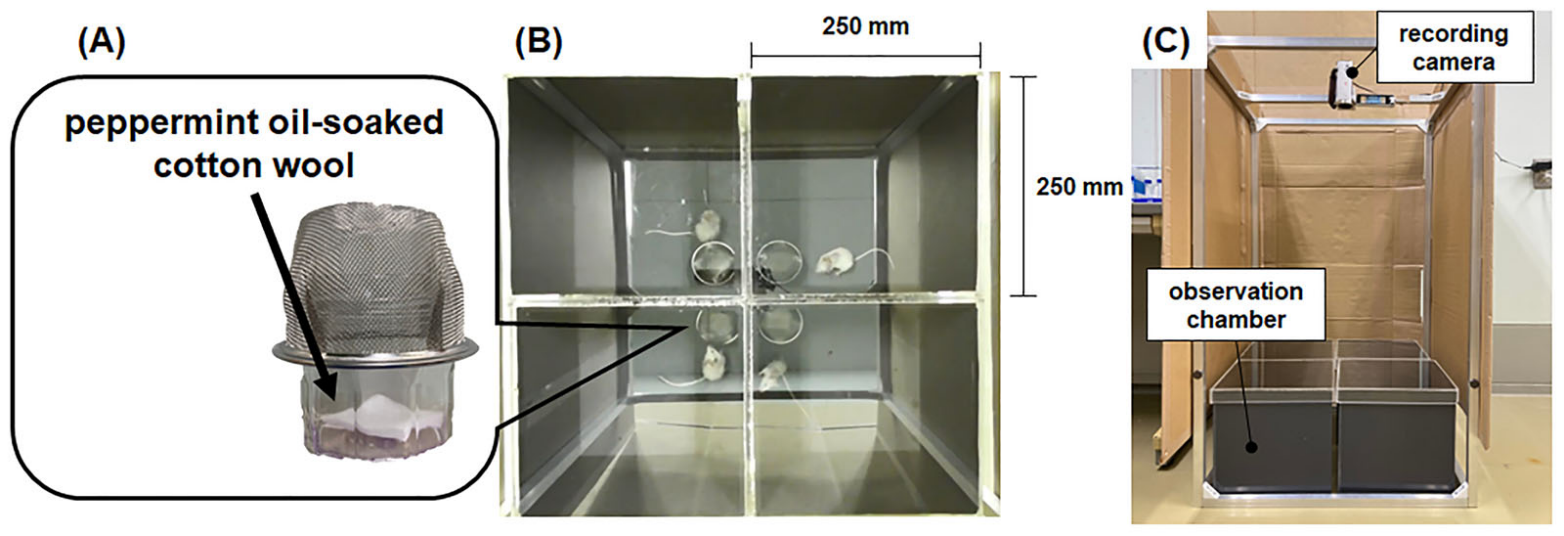
Experimental setup for the inhalation of peppermint essential oil (PEO) and recording of scratching behavior. (A) PEO‐soaked cotton wool placed in a glass container covered with a metallic wire mesh to avoid physical contact. (B) Actual photograph captured from above representing mice transferred to individual observation chambers made of dark gray opaque acrylic panel (250 × 250 × 250 mm). (C) Side view of the exposure apparatus. A recording camera was installed on top of the observation chambers.

PEO (batch number: 30K, TREE OF LIFE Co., Ltd., Tokyo, Japan) was diluted with double‐distilled water (ddw) to 2.5% (w/v). Diluted PEO (400 µL) was applied to a piece of cotton wool. The soaked cotton was placed in a glass container and covered with metallic wire mesh to avoid physical contact (Figure 1A). The PEO‐impregnated cotton was immediately placed in the corner of an observation chamber (Figure 1B). The chamber was closed with a clear acrylic lid to ensure the spread of PEO vapors. After 5 min, each mouse was transferred to one chamber. In the sham group, a constant volume (400 µL) of ddw was used instead of PEO.

### 2.5 Scratching Behavior

The behavior of the animals placed in the observation chamber was recorded. Scratching movements toward the nape of the neck were recorded using video cameras positioned above the behavioral observation chamber (Figure 1C). One scratching bout was defined as a single or series of scratching actions of the hind paws on the neck area that ended with either the paws returning to the floor mesh or the paws being licked (Liu et al. 2022). Although scratching behavior was recorded for 1 h, the total duration of scratching bouts was estimated only for 20 min, spanning from the 21st to 40th min from the start of the recording. As our pilot data indicated a strong correlation between the duration of scratching behavior in 1 h and this 20 min interval (Supplementary Figure 1), we adopted this simplified measurement approach. The scratching behavior was analyzed by a trained experimenter (SS) blinded to the experimental conditions by visual monitoring.

### 2.6 Experimental Design

**FIGURE 2:**
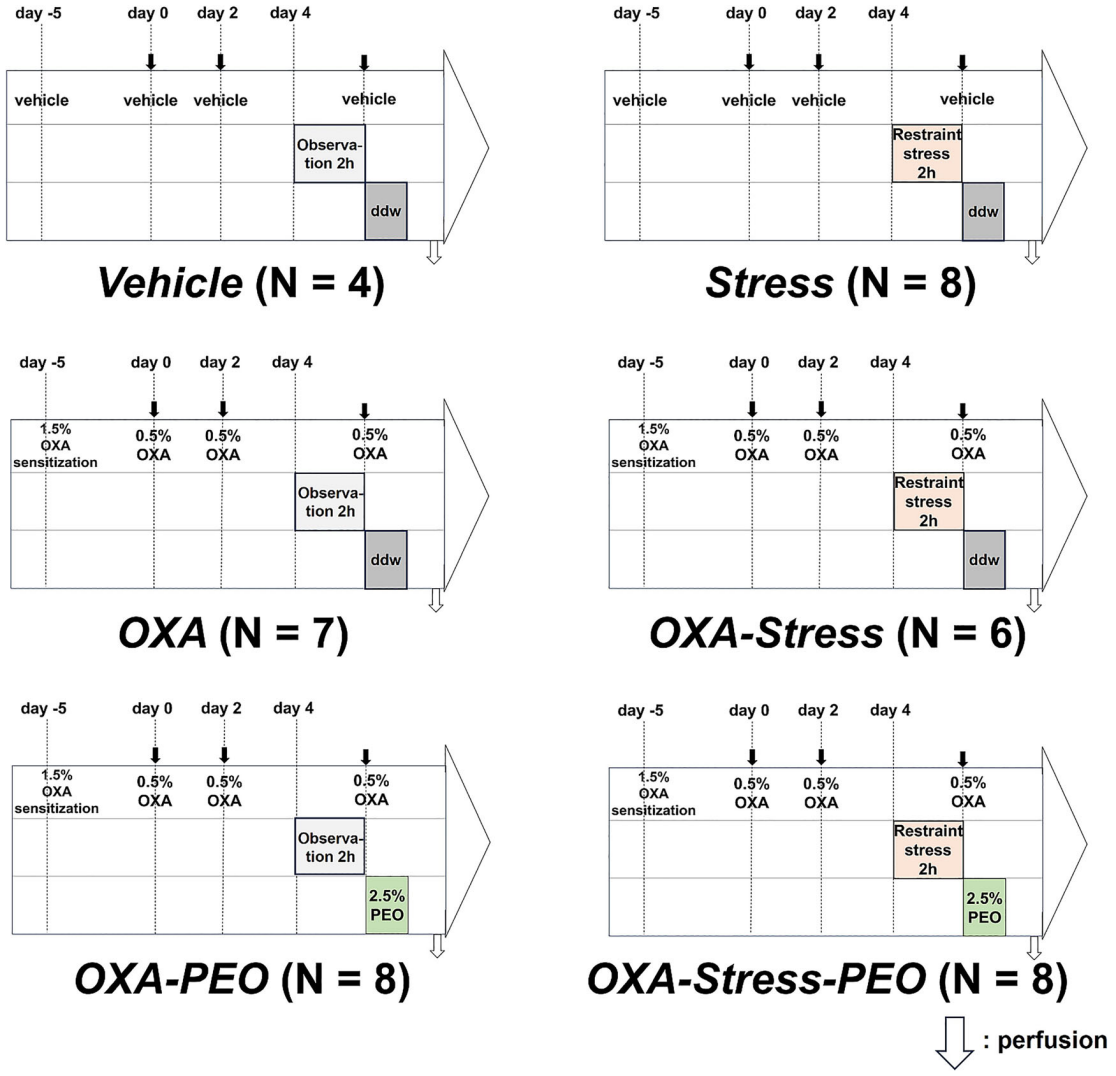
Experimental design. The animals were assigned to six groups. (1) *Vehicle* group (N = 4). Animals received a ddw patch on day −5 and repeated applications of the vehicle solution on days 0 and 2. On day 4, they were moved to a separate room and observed for 2 h. Following this, the vehicle solution was applied to the nape of the neck and the mice were transferred to the observation chamber to record the scratching behavior for 1 h; (2) *Stress* group (N = 8). Animals received a ddw patch on day −5 and repeated applications of the vehicle solution on days 0 and 2. On day 4, the animals were moved to a separate room and subjected to restraint stress for 2 h. Immediately after the termination of stress exposure, the vehicle solution was applied to the nape of the neck, and the mice were transferred to the observation chamber to record the scratching behavior for 1 h; (3) *OXA* group (N = 7). Animals were sensitized using 1.5% OXA solution on day −5, and dermatitis was induced by repeated applications of 0.5% OXA solution on days 0 and 2. On day 4, the animals were moved to a separate room and observed for 2 h. Subsequently, 0.5% OXA solution was applied to the nape of the neck, and the mice were transferred to the observation chamber to record the scratching behavior for 1 h; (4) *OXA‐Stress* group (N = 6). Animals were sensitized using 1.5% OXA solution on day −5, and dermatitis was induced by repeated applications of 0.5% OXA solution on days 0 and 2. On day 4, the animals were moved to a separate room and subjected to restraint stress for 2 h. Immediately after the termination of stress exposure, 0.5% OXA solution was applied to the nape of the neck, and the mice were transferred to the observation chamber to record the scratching behavior for 1 h; (5) *OXA‐PEO* group (N = 8). Animals were sensitized using 1.5% OXA solution on day −5, and dermatitis was induced by repeated applications of 0.5% OXA solution on days 0 and 2. On day 4, the animals were moved to a separate room and observed for 2 h. Following this, 0.5% OXA solution was applied to the nape of the neck, and the mice were transferred to the observation chamber filled with 2.5% PEO to record the scratching behavior for 1 h; and (6) *OXA‐Stress‐PEO* group (N = 8). Animals were sensitized using 1.5% OXA solution on day −5, and dermatitis was induced by repeated applications of 0.5% OXA solution on days 0 and 2. On day 4, the animals were moved to a separate room and subjected to restraint stress for 2 h. Immediately after the termination of stress exposure, 0.5% OXA solution was applied to the nape of the neck, and the mice were transferred to the observation chamber filled with 2.5% PEO to record the scratching behavior for 1 h.

The experimental design is illustrated in Figure 2. The animals were acclimated for one week after arrival and then randomly assigned to the following six experimental groups: (1) *Vehicle* group (N = 4): Animals were not exposed to any treatment from arrival; (2) *Stress* group (N = 8): Animals were subjected only to restraint stress; (3) *OXA* group (N = 7): Animals were subjected only to OXA‐induced dermatitis; (4) *OXA‐stress* group (N = 6): Animals were subjected to OXA‐induced dermatitis and restraint stress; (5) *OXA‐PEO* group (N = 8): Animals were subjected to OXA‐induced dermatitis and inhalation of PEO; and (6) *OXA‐Stress‐PEO* group (N = 8): Animals were subjected to OXA‐induced dermatitis and restraint stress, followed by PEO inhalation.

### 2.7 Brain Slice Preparation

One hour after recording, mice were anesthetized using a mixture of medetomidine hydrochloride (Nippon Zenyaku Kogyo, Tokyo, Japan), midazolam (Maruishi Pharmaceutical, Osaka, Japan), and butorphanol (Meiji Seika Pharma, Tokyo, Japan) and transcardially perfused with saline, followed by 100 mL of 4% ice‐cold paraformaldehyde in 0.1 M phosphate‐buffered saline (PBS; pH 7.4). The brain was extracted and post‐fixed overnight in the same fixative at 4°C and placed into graded concentrations of sucrose in PBS at 4°C. Coronal sections (30 µm) were cut through the PBN, PAG, VTA, and CeA according to the Mouse Brain Atlas (Franklin and Paxinos 2008) using a freezing microtome (CM1850, Leica Microsystems, Wetzlar, Germany). Sections were mounted on MAS‐coated slides, dried, and stored at −80°C. Due to technical difficulties, brain samples for immunohistochemistry analysis could not be obtained from two animals: one from the PBN in the *Stress* group and another from the VTA in the *OXA* group.

### 2.8 Immunohistochemistry

Brain sections were processed for c‐Fos immunoreactivity using an avidin‐biotin complex procedure. Every fourth brain section was selected for staining. Sections were treated with 0.3% H_2_O_2_ in methanol for 30 min to quench endogenous peroxidase activity, washed with PBS, and blocked with 5% normal goat serum in dilution buffer (1 × PBS containing 1% BSA, 0.4% Triton‐X 100, and 0.05% sodium azide) at 20°C–25°C for 1 h. Sections were incubated overnight at 4°C with rabbit anti‐c‐Fos primary antibodies (#2250, Cell Signaling Technology, Beverly, MA, USA, 1:2000 dilution in buffer). After rinsing, sections were incubated with biotinylated anti‐rabbit immunoglobulin G secondary antibody (BA‐1000, Vector Laboratories, Burlingame, CA, USA, 1:200 dilution) at 20–25°C for 2 h. After PBS rinses, sections were incubated with streptavidin‐horseradish peroxidase complex (P0397, Agilent Technologies, Santa Clara, CA, USA, 1:300 dilution) at 20°C–25°C for 1 h. After washing, slides were incubated with DAB (SK‐4100, Vector Laboratories) for 25 min and washed under tap water. After drying, slides were counterstained with hematoxylin, dehydrated using graded ethanol (70%, 80%, 90%, 95%, 99%, 100%, and 100%) for 5 min, and cleared with xylene three times. Coverslips were placed using Entellan New (#107961, Merck, Burlington, MA, USA) as a medium.

### 2.9 c‐Fos‐Positive Cell Counting

The sections were visualized using a digital microscope (DMD108, Leica Microsystems). Numbers of c‐Fos‐positive cells in the PBN, PAG, VTA, and CeA were quantified using the point tool in ImageJ software (version 1.53t, NIH, Bethesda, MD, USA) by a trained investigator (SS) who was blinded to the treatment by visual counting. Because the area in the brain region varies from section to section, the number of c‐Fos‐positive cells in each segmented brain region was divided by the area of the region estimated according to the Mouse Brain Atlas (Franklin and Paxinos 2008).

### 2.10 Statistical Analysis

Data were analyzed using StatView software Ver.5 (HULINKS, Tokyo, Japan). The Shapiro‐Wilk and Levene's tests were used to test the normality of data distribution and the homogeneity of variance in R software version 4.2.1 (Team 2022). The six experimental groups were separated into two subcategories for appropriate analysis: the first subcategory included *Vehicle*, *Stress*, *OXA*, and *OXA‐Stress* groups, and the second subcategory included *OXA*, *OXA‐Stress*, *OXA‐PEO*, and *OXA‐Stress‐PEO* groups. Two‐way analysis of variance (ANOVA) was performed to test for differences in the total duration of scratching bouts and quantification of c‐Fos‐positive cells in a part of the brain region, followed by Bonferroni/Dunn post‐hoc analysis for further examination of group differences with correction to adjust for multiple comparisons (α = 0.05/6 = 0.0083). For c‐Fos‐positive cells in PBN, VTA, and CeA in the first subcategory, a non‐parametric Kruskal‐Wallis test was used to compare multiple groups due to the variance's lack of homogeneity, followed by a post‐hoc Mann‐Whitney *U* test with correction to adjust for multiple comparisons (α = 0.05/6 = 0.0083). Regression analysis was performed to test for relationships between the total duration of scratching behavior as the independent variable and the number of c‐Fos‐positive cells per area in each brain region as the dependent variable. All data are expressed as the mean ± standard error of the mean (SEM). Statistical significance was set at *p* < 0.05.

## 3 Results

### 3.1 Total Duration of Scratching Behavior

**FIGURE 3:**
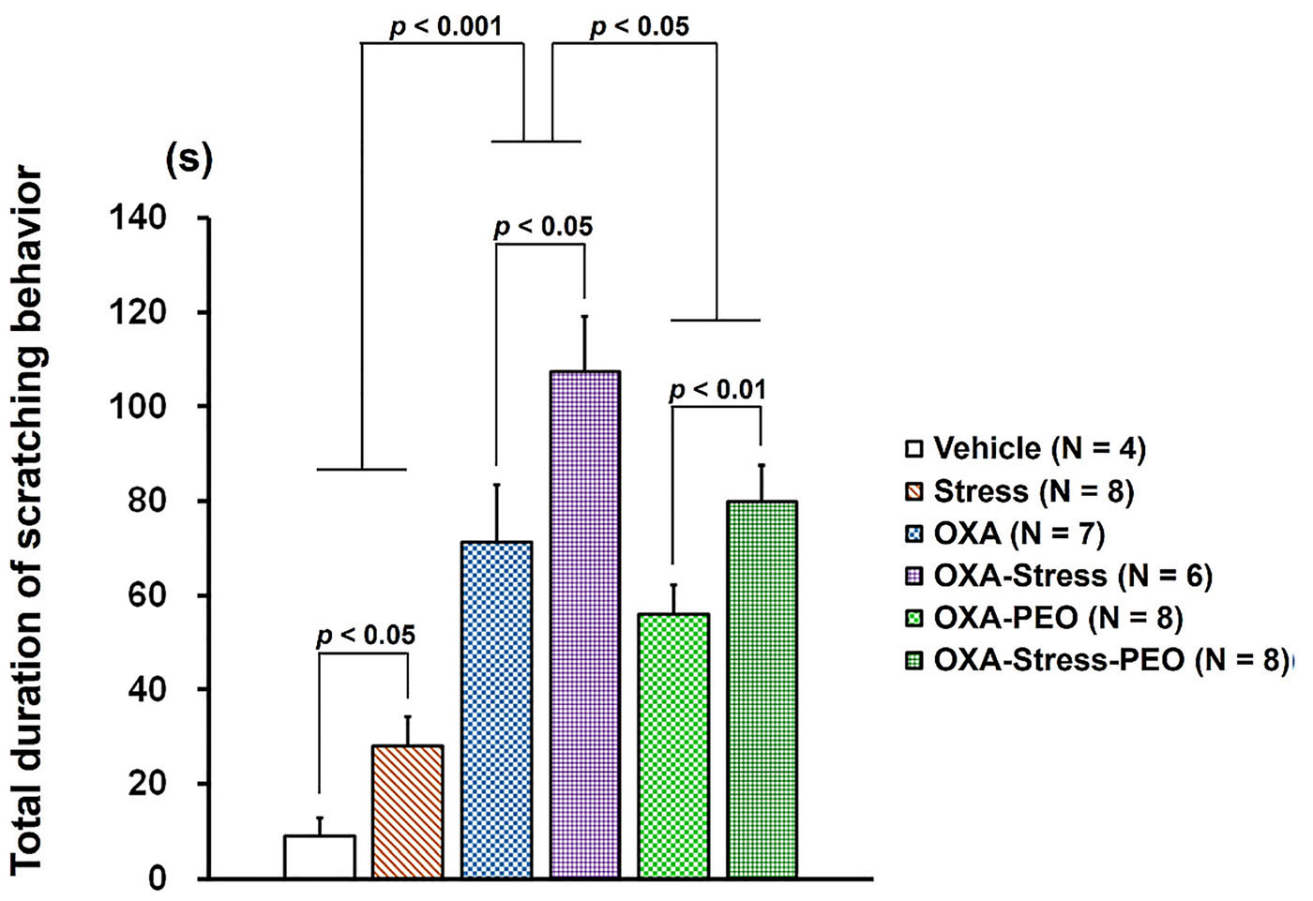
Effects of oxazolone‐induced dermatitis, acute stress, and inhalation of peppermint essential oil on the duration of scratching behavior.

Figure 3 shows the results of the scratching behavior analysis in the mice. A two‐way factorial ANOVA among first subcategory groups examined acute restraint stress effects on mice scratching behavior with/without OXA‐induced dermatitis. Results showed a significant increase (F_1, 21_ = 47.360, *p* < 0.001) in scratching bout duration in mice with OXA‐induced dermatitis (*OXA* and *OXA‐Stress* groups) versus vehicle‐treated mice (*Vehicle* and *Stress* groups). Statistical analysis revealed that scratching bout duration in stress‐exposed mice (*Stress* and *OXA‐Stress* groups) was significantly greater than non‐stress‐matched controls (*Vehicle* and *OXA* groups) (F_1, 21_ = 7.208, *p* < 0.05). Two‐way factorial ANOVA among second subcategory groups, examining PEO inhalation effects on scratching during OXA‐induced dermatitis under stress conditions, showed a significant increase (F_1, 25_ = 9.987, *p* < 0.01) in scratching bout duration of stress‐loaded mice (*OXA‐Stress* and *OXA‐Stress‐PEO* groups) versus non‐stressed mice (*OXA* and *OXA‐PEO* groups). The scratching bout duration in PEO‐inhaled mice (*OXA‐PEO* and *OXA‐Stress‐PEO* groups) was significantly lower than in ddw‐exposed matching groups (*OXA* and *OXA‐Stress* groups) (F_1, 25_ = 5.055, *p* < 0.05). No significant interaction between stress and PEO inhalation was observed.

### 3.2 Number of c‐Fos‐Positive Cells in Brain Regions Associated With Itch

**FIGURE 4:**
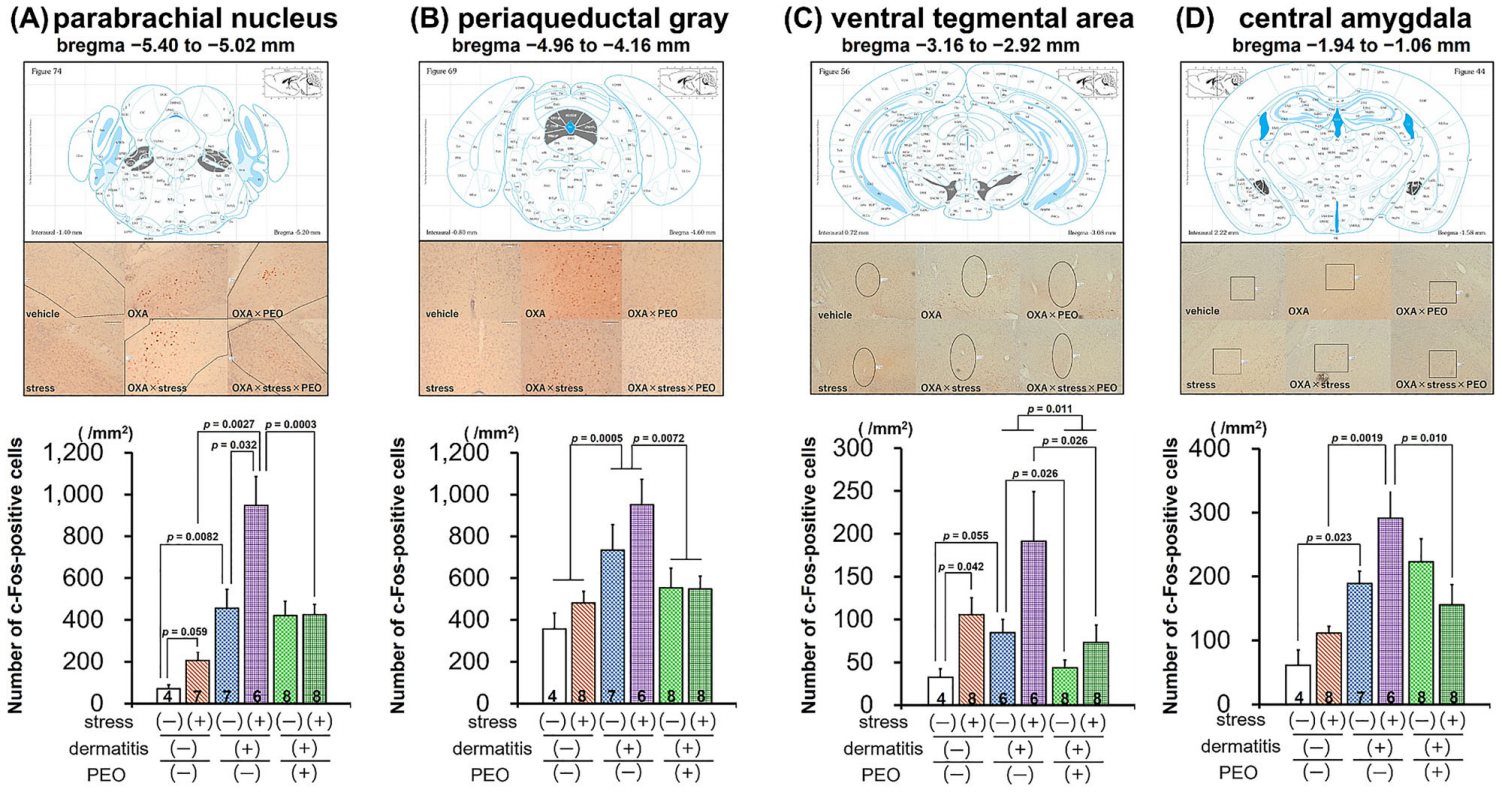
Effects of oxazolone‐induced dermatitis, acute stress, and inhalation of peppermint essential oil on the number of c‐Fos‐positive cells per unit area. In each panel, the upper subpanel represents each brain region with the areas painted in gray (coronal section illustration sourced from Franklin and Paxinos 2008). The middle subpanel represents the photomicrographs of c‐Fos‐positive cells (80× magnification). The lower subpanel represents the quantification of c‐Fos‐positive cells in each brain region. (A) parabrachial nucleus (PBN), (B) periaqueductal gray (PAG), (C) ventral tegmental area (VTA), and (D) central amygdala (CeA). The numbers displayed in the bar graph represent the sample size of each group.

Figure 4 shows the number of c‐Fos‐positive cells per unit area (counts/mm^2^) in each brain region. The Kruskal‐Wallis test revealed statistically significant differences in c‐Fos‐positive cell numbers in the PBN among *Vehicle*, *Stress*, *OXA*, and *OXA‐Stress* groups (H = 16.77, *p* < 0.001). A post‐hoc Mann‐Whitney *U* test showed that the number of c‐Fos positive cells in the PBN was significantly greater in the *OXA* group compared to the *Vehicle* group (z = −2.65, *p* = 0.0082). The *OXA‐Stress* group exhibited a significantly higher number of c‐Fos positive cells compared to the *Stress* group (z = −3.00, *p* = 0.0027). Two‐way factorial ANOVA among *OXA*, *OXA‐Stress*, *OXA‐PEO*, and *OXA‐Stress‐PEO* groups revealed significant effects of acute stress (F_1, 25_ = 8.40, *p* < 0.01), PEO inhalation (F_1, 25_ = 10.73, *p* < 0.01), and stress × PEO interaction (F_1, 25_ = 8.15, *p* < 0.01). Post‐hoc analysis showed significantly fewer c‐Fos‐positive cells in the PBN in the *OXA‐Stress‐PEO* group compared to the *OXA‐Stress* group (*p* = 0.0003) but not the *OXA* group.

Two‐way factorial ANOVA among *Vehicle*, *Stress*, *OXA*, and *OXA‐Stress* groups showed OXA‐induced dermatitis significantly affected c‐Fos‐positive cells in the PAG region (Figure 4B) (F_1, 21_ = 16.75, *p* < 0.001). Analysis of *OXA*, *OXA‐Stress*, *OXA‐PEO*, and *OXA‐Stress‐PEO* groups revealed PEO inhalation significantly affected cell numbers (F_1, 25_ = 8.57, *p* < 0.01).

The Kruskal‐Wallis test revealed statistically significant differences in c‐Fos‐positive cells in the VTA among the *Vehicle*, *Stress*, *OXA*, and *OXA‐Stress* groups (H = 8.14, *p* < 0.05). A post‐hoc Mann‐Whitney *U* test showed that the number of c‐Fos positive cells in the VTA was higher in the *Stress* group compared to the *Vehicle* group; however, this difference was not statistically significant (z = −2.04, *p* = 0.042). Two‐way factorial ANOVA of *OXA*, *OXA‐Stress*, *OXA‐PEO*, and *OXA‐Stress‐PEO* groups showed that acute stress (F_1, 24_ = 5.64, *p* < 0.05) and PEO inhalation (F_1, 24_ = 7.68, *p* < 0.05) significantly affected cell numbers.

The Kruskal‐Wallis test revealed statistically significant differences in c‐Fos‐positive cells in the CeA among the *Vehicle*, *Stress*, *OXA*, and *OXA‐Stress* groups (H = 16.68, *p* < 0.001). A post‐hoc Mann‐Whitney *U* test showed that the number of c‐Fos positive cells in the CeA was significantly higher in the *OXA‐Stress* group compared to the *Stress* group (z = −3.10, *p* = 0.0019). Additionally, the *OXA* group did not show a significantly higher number of c‐Fos positive cells compared to the *Vehicle* group (z = −2.27, *p* = 0.023). Two‐way factorial ANOVA of *OXA*, *OXA‐Stress*, *OXA‐PEO*, and *OXA‐Stress‐PEO* groups showed that cell numbers were significantly affected by stress × PEO interaction (F_1, 25_ = 6.31, *p* < 0.05). Post‐hoc analysis showed fewer c‐Fos‐positive cells in the CeA in the *OXA‐Stress‐PEO* group compared to the *OXA‐Stress* group, but the difference was not statistically significant (*p* = 0.010).

### 3.3 Relationship Between the Total Duration of Scratching Behavior and the Number of c‐Fos‐Positive Cells in the Brain Regions Associated With Itching

**FIGURE 5:**
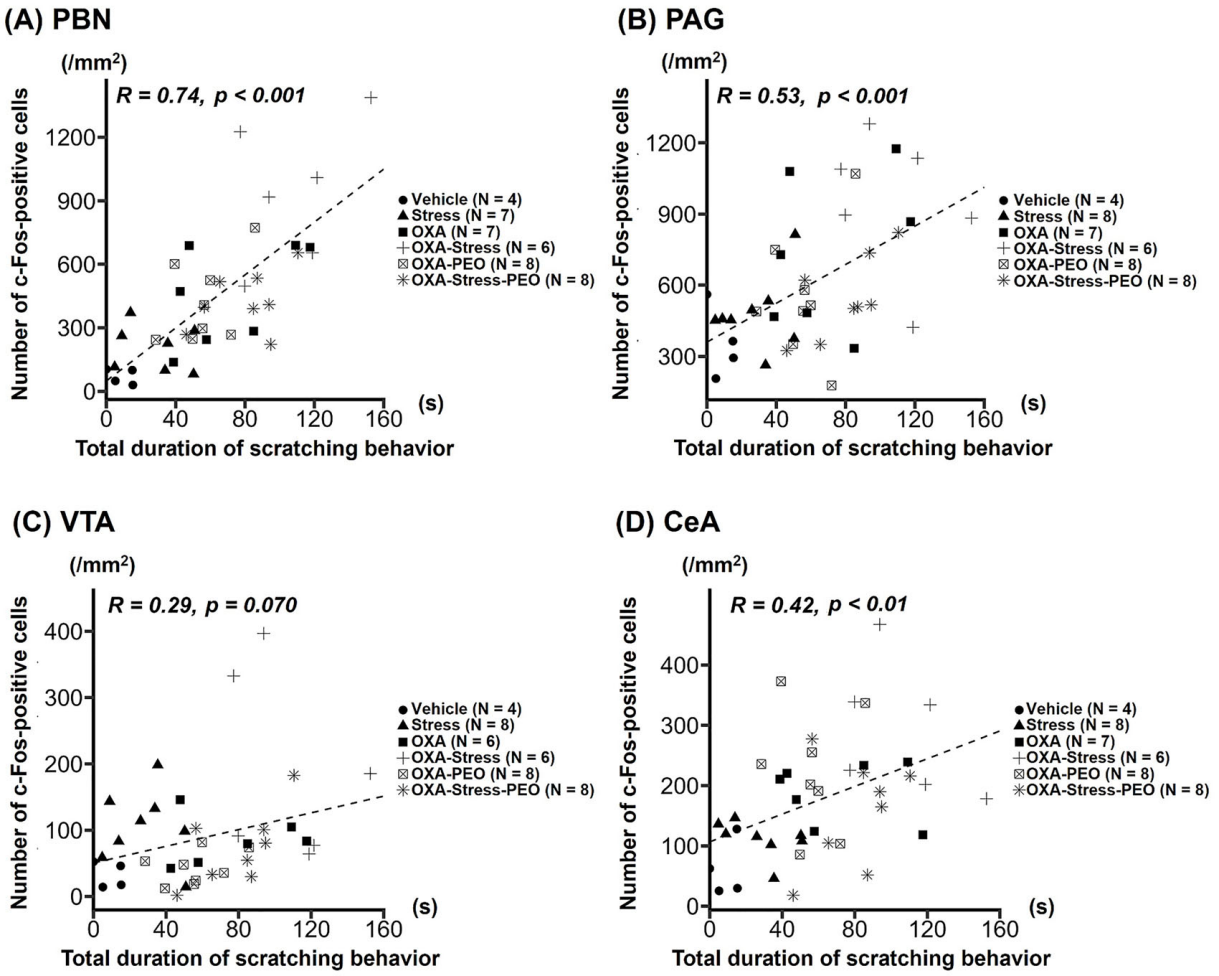
Relationship between the total duration of scratching behavior and the number of c‐Fos‐positive cells in brain regions associated with itching. (A) parabrachial nucleus (PBN), (B) periaqueductal gray (PAG), (C) ventral tegmental area (VTA), and (D) central amygdala (CeA).

Figure 5 shows the relationship between total scratching duration and the number of c‐Fos‐positive cells in the four brain regions. Significant positive relationships were observed in all brain regions, except the VTA. The c‐Fos‐positive cells in the PBN were strongly related to the total duration of scratching behavior (Figure 5A).

## 4 Discussion

The present study yielded two major findings. First, PEO inhalation alleviated increased total scratching duration induced by the combination of OXA application and acute restraint stress. Second, c‐Fos‐positive cells in brain regions associated with itch positively correlated with pruritic responses.

Although topical PEO or menthol reduces itch perception (Herro and Jacob 2010; Patel et al. 2007), to our knowledge, this is the first report of PEO inhalation alleviating itch‐induced scratching behavior under stress conditions. The anti‐pruritic effect of menthol, a major PEO component, has been linked to stimulation of transient receptor potential melastatin (TRPM) 8 in peripheral sensory neurons, producing cooling sensations (Mahmoud, Soares, and Yosipovitch 2022). Nakashimo et al. (2010) showed TRPM8 expression in mouse olfactory epithelium, and Shakhawat et al. (2014) found PEO inhalation elicited olfactory responses. Beukema et al. (2018) showed increased c‐Fos‐positive cells in the anterior piriform cortex after menthol exposure, which was inhibited in TrpM8‐knockout mice. The anterior piriform cortex receives olfactory information and transmits it to brain regions affecting emotion (Soudry et al. 2011). Matsukawa et al. (2022) showed this region has an inhibitory system for odor‐related stress responses. These findings suggest that PEO inhalation may increase anterior piriform cortex activity through TRPM8 stimulation, thereby reducing neuronal activity in stress‐responsive regions. Future studies should investigate whether PEO inhalation reduces pruritic behavior in mice treated with TRPM8 antagonists or in TrpM8‐knockout mice.

The current study also found that acute stress increased scratching behavior in mice regardless of OXA‐induced dermatitis. While acute stress effects on itching are not fully understood, one study showed increased scratching behavior in mice (Cho et al. 2024). Other studies reported stress‐induced decreases in scratching bouts (Spradley, Davoodi, Carstens, and Carstens 2012; Wang et al. 2022). This inconsistency may be due to differences in stress exposure duration and evaluation timing. Studies showing reduced itching used short durations of forced swim stress and evaluated responses within 5‐10 min. Cho et al. (2024) found 2 h of immobilization stress increased scratching in mice with dermatitis. These findings suggest immediate short‐term stress induces antinociception, while prolonged stress enhances pruritic behavior. Mochizuki et al. (2019) showed 15‐min acute stress increased scratching in atopic dermatitis patients. Theoharides et al. (1998) reported elevated corticotropin‐releasing hormone may activate skin mast cells, causing pruritus. However, acute stress effects were not observed in healthy participants (Mochizuki et al. 2019). Furthermore, it cannot be ruled out that stress‐induced increase in scratching behavior may be affected by other behavioral changes induced by acute restraint stress, such as grooming, locomotor activity, and anxiety‐like behaviors in healthy rodents and mice with dermatitis. Future studies should examine whether PEO inhalation attenuates stress‐induced pruritic response and other behavioral alterations in healthy humans and rodents.

We observed increased c‐Fos‐positive cells in the PBN, PAG, VTA, and CeA during OXA‐induced dermatitis. The PBN is central to itch sensation (Mu et al. 2017; Ren et al. 2023). Gao et al. (2019) reported PAG neuronal activity involvement in itch‐induced scratching. The VTA facilitates acute and chronic itching (Su et al. 2019). The CeA plays a role in the affective component of pain and pruritus (Neugebauer 2015; Sanders et al. 2019). These studies showed increased c‐Fos‐positive cells in these regions under pruritogen‐induced itch. Our findings from a hapten‐induced model align with these results. Acute restraint stress increased c‐Fos‐positive cells in the PBN, VTA, and CeA. Godoy et al. (2018) noted that restraint stress activates VTA and CeA neurons. Three days of restraint stress increased c‐Fos‐positive cells in the PBN and CeA (Xu et al. 2023). PBN‐to‐CeA excitatory projections stimulate pain processing (Cai et al. 2018; Jaramillo et al. 2021), while CeA‐to‐PBN pathways reduce itch sensation (Hogri et al. 2022; Raver et al. 2020). PBN neurons innervating the substantia nigra inhibit VTA dopaminergic activity, potentially promoting aversive behaviors (Yang et al. 2021). These findings suggest complex, bidirectional neuronal networks between brain regions involved in itch processing and stress response. Which brain area is most important for pruritic behavior under stress remains unclear. Although a strong correlation between PBN c‐Fos‐positive cells and scratching behavior implies that the PBN is critical for pruritic responses induced by acute stress and experimental dermatitis, the functional link between alterations in scratching behavior and the number of c‐Fos‐positive cells in the PBN remains unclear, as c‐Fos‐immunoreactive cells serve as an indirect marker of neuronal activity. Future research should investigate the effects of activating or inhibiting the PBN on stress‐induced scratching behavior, utilizing electrophysiology, calcium imaging, region‐specific chemical lesions, or chemogenetic manipulation. Moreover, noxious stimuli and descending neural activation centrally suppress ascending itch transmission in the spinal cord (Follansbee et al. 2022; Gotoh et al. 2011; Mochizuki et al. 2003), suggesting that the descending itch inhibitory system may play a role in mitigating scratching behavior induced by inhalation exposure to PEO. Because the descending itch inhibitory system comprises the PAG, locus coeruleus, or rostral ventromedial medulla, how the activity of these brain regions changes following PEO inhalation merits further investigation.

The current study has several limitations. First, the limited sample size and the exclusive use of female mice are likely to have introduced bias into the research findings. Second, we did not include a healthy PEO inhalation exposure group, and PEO exposure was only at a single concentration. An odor preference test showed that menthol is aversive to mice (Saraiva et al. 2016). Given that aversive odors elicit anxiety/fear‐related behaviors (Blanchard et al. 2003), inappropriate concentrations of PEO inhalation might induce stress responses. Future studies should assess behavioral properties and glucocorticoid levels after PEO inhalation in a dose‐dependent manner. Third, the route through which odor information reaches the brain remains unclear. Aromatic molecules bind to olfactory receptors in the olfactory epithelium, and odorant information is sent to the olfactory bulb and secondary structures (Gottfried 2010). Future studies should use anosmic mice with nasal cavity irrigation (Chioca, Antunes, Ferro, Losso, and Andreatini 2013) to confirm olfaction's role in PEO's anti‐pruritic effects. In addition, volatile compounds are absorbed via respiration, distributed into blood, and transported to the brain (Kasuya, Iida, Ono, Satou, and Koike 2015; Satou et al. 2017). Feng et al. (2019) reported the pharmacokinetic profile of menthol after a single inhalation by quantifying the plasma menthol concentration. Although we could not ascertain whether the concentration and flow rate of PEO vapor were consistent, future research should verify if all mice were equally exposed by quantifying the concentration of PEO metabolites, such as menthol, within their organs. Fourth, the OXA‐induced dermatitis was relatively mild due to short‐term intervention. While chronic hapten exposure can lead to atopic dermatitis‐like lesions (Smith et al. 2019), short‐term OXA application causes allergic contact dermatitis‐like conditions. Since itching is primary in many diseases, future research must clarify whether pruritus from various pathological conditions can be regulated by stress exposure or PEO inhalation. Finally, the analysis of scratching behavior was confined to the middle 20 min of each one‐hour recorded video. As illustrated in Supplementary Figure 1, preliminary investigations revealed a strong correlation between scratching behavior during this middle period and the total scratching behavior over the entire hour. However, this correlation has not been assessed under conditions of stress load or PEO inhalation. Consequently, further investigation is warranted to determine the appropriateness of reducing the analysis time for scratching behavior across various experimental conditions.

## 5 Conclusion

The current study demonstrated that pruritus caused by OXA‐induced dermatitis is exacerbated by acute restraint stress and attenuated by the inhalation of PEO, which is associated with changes in neuronal activity in itch‐related brain regions.

## Author Contributions

**Shotaro Shoji**: conceptualization, data curation, formal analysis, investigation, methodology, visualization, writing – original draft, writing – review and editing. **Yusuke Murata**: conceptualization, data curation, formal analysis, funding acquisition, investigation, methodology, project administration, supervision, validation, visualization, writing – original draft, writing – review and editing. **Hikari Ikeda**: formal analysis, investigation. **Reika Sakai**: formal analysis, investigation. **Yuka Chijiiwa**: formal analysis, investigation. **Masayoshi Mori**: supervision, visualization. **Munechika Enjoji**: conceptualization, supervision, writing – review and editing.

## Funding

This work was supported by Leave a Nest (DUSKIN Research and Development 57th award to Yusuke Murata).

## Ethics Statement

All animal experiments were performed in accordance with the regulations established by the Experimental Animal Care and Use Committee of Fukuoka University, following the universal principles of laboratory animal care (approval numbers: 2204003 and 2304004; dates of approval: 4/19/2022 and 4/26/2023, respectively).

## Conflicts of Interest

None of the authors have a conflict of interest to disclose

## Supporting information



Supplementary Figure 1. Our pilot data indicated a relationship between the total duration of scratching behavior during the total time span of 60 min and during the specified 20 min interval within this time span.

## Data Availability

Data will be made available on request.
