# Supplementary material for: Single Inhalation of Peppermint Essential Oil Alleviates Acute Restraint Stress‐Exacerbated Itch in Oxazolone‐Induced Mild Dermatitis: Correlations With Brain Neuronal Activity in Female BALB/c Mouse
Source: Brain Behav. 2025 Nov 17;15(11):e71072. doi: 10.1002/brb3.71072 (PMC12623463; doi:10.1002/brb3.71072)
Supplement: Supplementary file 1 — Supplementary Figure 1. Our pilot data indicated a relationship between the total duration of scratching behavior during the total time span of 60 min and during the specified 20 min interval within this time span. [file BRB3-15-e71072-s001.docx]

**
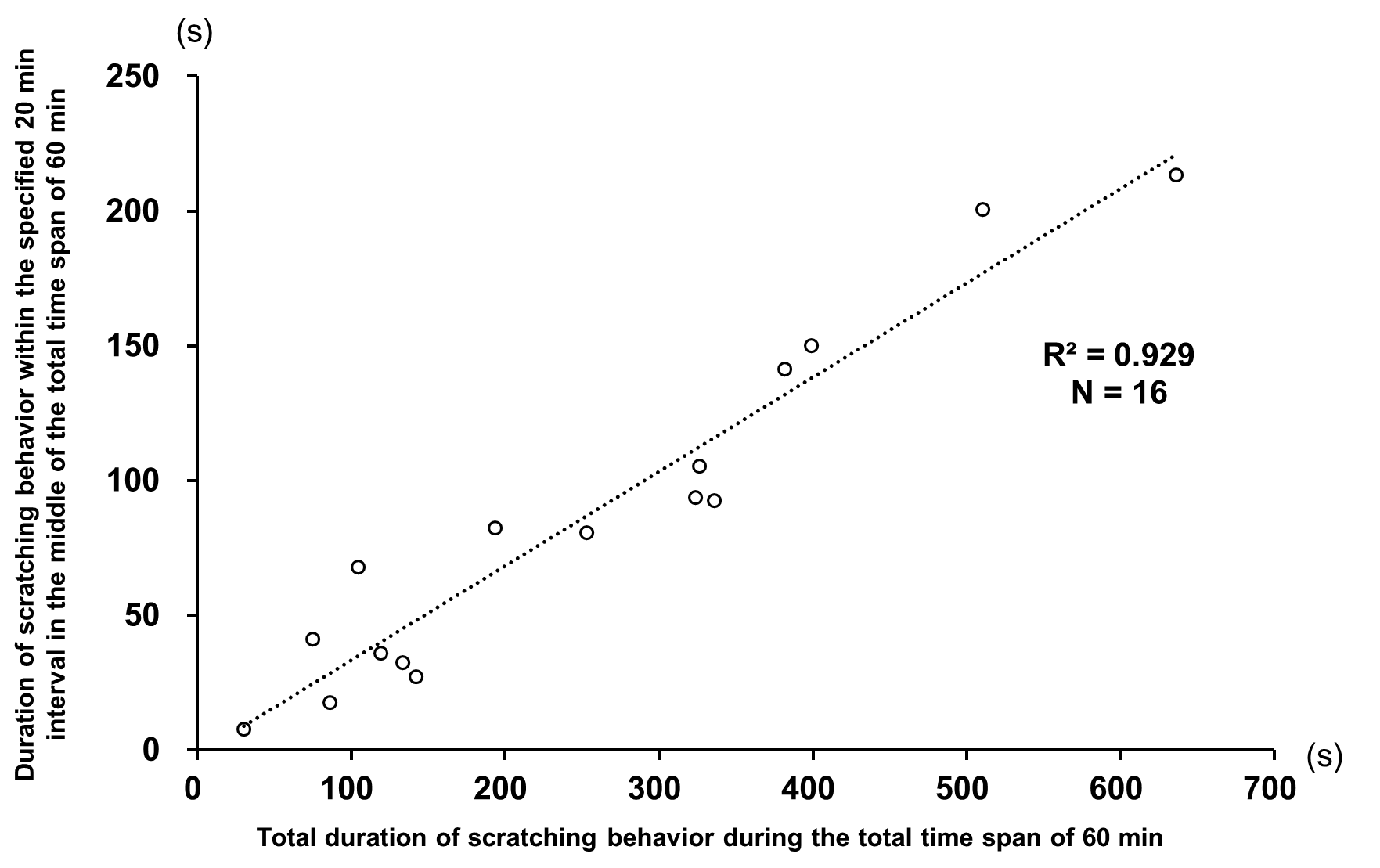
Supplementary Data**

**Supplementary Figure 1.** Our pilot data indicated a relationship between the total duration of scratching behavior during the total time span of 60 min and during the specified 20 min interval within this time span. In the pilot study, the scratching behavior was assessed using similar techniques for two experimental groups: vehicle-treated group (N = 4) and intermittent OXA-induced dermatitis group (N = 12).
